# Identification of Germline Mismatch Repair Gene Mutations in Lung Cancer Patients With Paired Tumor-Normal Next Generation Sequencing: A Retrospective Study

**DOI:** 10.3389/fonc.2019.00550

**Published:** 2019-06-26

**Authors:** Sibo Sun, Yiqian Liu, Ann-Kathrin Eisfeld, Fuxi Zhen, Shidai Jin, Wen Gao, Tongfu Yu, Liang Chen, Wei Wang, Wei Chen, Mingming Yuan, Rongrong Chen, Kai He, Renhua Guo

**Affiliations:** ^1^Department of Oncology, The First Affiliated Hospital of Nanjing Medical University, Nanjing, China; ^2^Departments of Internal Medicine and Pathology, The Ohio State University Wexner Medical Center, Columbus, OH, United States; ^3^Department of Cardiothoracic Surgery, The First Affiliated Hospital, Nanjing Medical University, Nanjing, China; ^4^Department of Radiology, The First Affiliated Hospital of Nanjing Medical University, Nanjing, China; ^5^Department of Thoracic Surgery, The First Affiliated Hospital of Nanjing Medical University, Nanjing, China; ^6^Department of R&D, Geneplus-Beijing Institute, Beijing, China

**Keywords:** Lynch syndrome, lung cancer, next-generation sequencing, mismatch repair gene, cancer risks

## Abstract

**Background:** Paired tumor-normal targeted next-generation sequencing (NGS) is primarily used to identify actionable somatic mutations, but can also detect germline variants including pathogenic germline mutations in DNA mismatch repair (MMR) genes that underlie Lynch syndrome. In the present study we examined paired NGS data from lung cancer patients to identify germline mutations in MMR genes. As lung cancer is not one of the recognized Lynch syndrome-associated neoplasms, we also investigated whether these lung cancer cases are due to Lynch syndrome or are instead sporadic cancers occurring in Lynch syndrome patients.

**Methods:** A retrospective study of 1,179 lung cancer patients with available paired NGS data was performed to identify germline mutations in the MMR genes *MLH1, MSH2, MSH6*, and *PMS2*, and evaluate tumor mutation burden (TMB). Microsatellite instability (MSI) testing was done on select cases with MMR gene mutations by either NGS or PCR/capillary electrophoresis approach. Immunohistochemistry (IHC) for MMR proteins was performed in select patients.

**Results:** Pathogenic or likely-pathogenic germline mutations in *PMS2, MSH2*, or *MSH6* were detected in 0.5% (6/1,179) of lung cancer patients; three of the patients had a family history of colon or gastric cancer. The median age at diagnosis of these cases was 68.5 years old. None of these six patients exhibited MSI or loss of MMR protein expression. Among them, no second hit somatic mutations in MMR genes (including single-nucleotide variants, small insertions or deletions and copy number alterations) were detected, and the median TMB was 4.5 muts/MB. Subsequent genetic testing of family members identified new Lynch syndrome cases in two first-degree relatives.

**Conclusion:** These data imply that lung cancers in Lynch syndrome patients are unrelated to the underlying Lynch syndrome diagnosis and occur spontaneously. Nonetheless, paired tumor-normal NGS can identify germline mutations to help reveal Lynch syndrome in cancer patients. This has important implications for cancer screening and risk reduction in these patients and their families.

## Introduction

Recent studies have identified genes in which germline mutations are associated with lung cancer, even in non-smokers, including *EGFR, BRCA2, TP53*, and others (1, 2). However, little is known about the relationship between lung cancer risk and germline mutations that underlie common hereditary cancer syndromes, such as Lynch syndrome, which is caused by mutations in DNA mismatch repair (MMR) genes including *MSH2, MSH6, MLH1*, and *PMS2*. Individuals harboring germline loss-of-function variants in MMR genes have an elevated risk of specific cancer types, especially colorectal, endometrial, and gastric carcinomas. However, a recent pan-cancer study reported that germline gene mutations in *MSH6* were present in ~1% (2/191) of lung cancers patients (3). Given that lung cancer is not frequently reported in Lynch syndrome patients, the relationship between lung cancer risk, and germline MMR mutations requires further exploration.

Loss of MMR protein function results in the accumulation of mutations, especially insertions and deletions (indels) in repetitive sequences called microsatellites, which is termed microsatellite instability (MSI). MSI can be used as a surrogate to infer loss of MMR protein function, and is routinely assessed using PCR of select microsatellite loci followed by capillary electrophoresis. In recent years NGS-based methods of detecting MSI have also become commonplace (4). Notably, MSI requires inactivation of both alleles of an MMR gene, which occurs through bi-allelic loss-of-function mutations, epigenetic silencing or copy number deletion (“second hits”). In addition to MSI assessment, loss of MMR protein expression can be directly examined in tumors using immunohistochemistry (IHC).

Comprehensive NGS-based molecular testing is becoming more common in cancer patient care. In clinical practice, matched tumor-normal sequencing is essential for the identification of somatic mutations and aids in selecting patients for targeted therapies based on the actionable cancer driver mutations. An additional benefit of tumor-normal sequencing is the *de facto* screening for germline variants that cause heritable disorders, which provides useful information to patients and their families without extra testing costs. If adequate DNA is not available from the tumor biopsy, sequencing circulating tumor DNA (ctDNA) can also uncover actionable mutations (5, 6). To investigate the prevalence of Lynch syndrome in unselected lung cancer patients without known heritable disorders, we retrospectively examined NGS testing results from 1,179 lung cancer patients for germline loss-of-function mutations in MMR genes. For patients harboring germline MMR gene mutations, we evaluated their somatic mutation landscape including TMB, MSI status, MMR protein expression, and clinical and pedigree characteristics when possible based on material or information availability.

## Results

### Retrospective Assessment of Germline MMR Gene Mutations in Lung Cancer Patients

**Figure 1:**
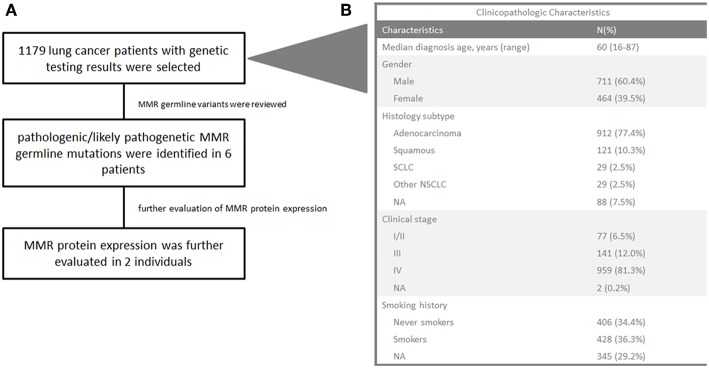
Flowchart of the study **(A)** and clinicopathological characteristics of 1,179 lung cancer patients **(B)**. SCLC, small-cell lung cancer; NA, not available. Other NSCLC refers to dispersive pathological subtypes including adenosquamous carcinoma, sarcomatoid carcinoma, lymphoepithelioma-like carcinoma, mucoepidermoid carcinoma, and similar subtypes.

To assess the prevalence of germline MMR mutations in lung cancer patients, we retrospectively reviewed the mutation profiles of 1,179 lung cancer patients who underwent genetic testing of 1,021 cancer-related genes at Geneplus-Beijing Institute (Beijing, China) between 2017 and 2018 (Supplementary Table S1, Figure 1A). The clinicopathological characteristics of these patients are summarized in Figure 1B. The median age at diagnosis was 60 years (range 16–87); 464 (39.5%) patients were female, 912 (77.4%) patients were diagnosed as lung adenocarcinoma, and 121 (10.3%) patients were diagnosed as lung squamous cell carcinoma. The majority of patients, 959 (81.3%), were at stage IV.

Targeted NGS was used to detect somatic mutations in cancer related genes (Supplementary Table S2) and germline variants in DNA MMR genes (Supplementary Table S3). Pathogenic or likely-pathogenic germline mutations in the assessed MMR genes were detected in 0.5% (6/1,179) of patients (*MSH2*, one patient; *PMS2*, two patients; *MSH6*, three patients) (Table 1, Supplementary Table S3). No *MLH1* germline mutations were identified in our cohort. The median age at diagnosis of these six patients was 68.5 years old, which was significantly older than that of the entire cohort (*p* = 0.0355). Three were female and two were smokers. None of these six patients had a personal history of cancer. Case 1 and Case 4 had a family history of colon cancer in their first-degree relatives. Additionally, the brother of Case 6 was previously diagnosed with gastric cancer, which is a Lynch syndrome-associated malignancy (7).

**Table 1:** Baseline characteristics and genetic testing results of lung cancer patients with germline MMR mutations.

**Case**	**Age range at diagnosis**	**Smoking status**	**Family history**	**Histological subtype**	**Sample type**	**Germline mutation**	**TMB (muts/Mb)**	**MSI**	**MMR expression**	**Genes with somatic mutations**
1	60–65	NS	Mother, colon cancer	ADC	PB	*MSH2* NM_000251.2, c.340delG, p.E114Rfs^*^60	1	N/A	Intact	*MAP2K2, GNAS*
2	70–75	NS	No	ADC	PB	*PMS2* NM_000535.5, c.943C>T, p.R315^*^	1	MSS (PCR)	Intact	*PTCH1*
3	70–75	NS	No	ADC	FFPE	*PMS2* NM_000535.5 c.1053delG, p.L351Ffs^*^5	5	MSS (NGS)	N/A	*GNAS, EGFR, MTOR, CUL3, CREBBP, FGFR4, ABCB1*
4	55–60	NS	Mother, colon cancer	ADC	FFPE	*MSH6* NM_000179.2 c.3118T[3>1], p.F1040^*^	4	MSS (NGS)	N/A	*MLL3, EGFR, TP53, RB1, NXF5, CBL*
5	65–70	S	No	NSCLC	FFPE	*MSH6* NM_000179.2 c.4001G>A p.R1334Q	6	MSS (NGS)	N/A	*IGF1R, TP53, ARID2, XRCC3, MET, SLC34A2, LRP1B, DICER1*
6	75–80	S	Brother, gastric cancer	SCC	FFPE	*MSH6* NM_000179.2 c.2552_2553dupGC, p.K852Afs^*^17	8	MSS (NGS)	N/A	*ALK, LRP1B, TP53, DNMT3A, HRAS, DDR2, NTM, TCF7L2, POLE*

*NS, never-smoker; S, smoker; ADC, adenocarcinoma; NSCLC, non-small-cell lung cancer, SCC, squamous-cell carcinoma; PB, peripheral blood; FFPE, formalin fixation and paraffin embedding tissue; TMB, tumor mutation burden, MSI, microsatellite instability; MSS, microsatellite stable; N/A, not available*.

We then investigated whether the detected germline MMR gene mutations led to MMR deficiency (MSI or loss of MMR protein expression) in these lung cancer cases. Notably, no somatic “second-hit” mutations were detected in these tumors (Table 1). The MSI status of Cases 3–6 was assessed using MSIsensor (v0.5) with NGS data of tumor tissue (8), and all four cases were microsatellite stable (MSS). PCR-based MSI detection was performed on tumor tissue from Case 2, and likewise this sample was MSS. IHC was used to detect the expression of MLH1, MSH2, MSH6, and PMS2 in Cases 1 and 2, and both samples showed intact expression for the four MMR proteins (Figure S1). Therefore, none of the six patients with germline MMR gene mutations showed evidence for somatic inactivation of MMR genes.

Finally, we evaluated TMB in the entire lung cancer patient set, to determine if the six cases with germline MMR gene mutations showed elevated TMB. TMB-high was classified as ≥9 muts/MB using the top quartile threshold of 2,000 samples from the Geneplus database (9, 10). TMB ranged from 1 to 8 muts/MB, with a median of 4.5 muts/MB in the six patients with germline MMR gene mutations, while TMB in the entire cohort ranged from 1 to 80 muts/MB, with a median of 7 muts/MB; no significant difference was observed (*p* = 0.0698).

## Case Presentations

**Figure 2:**
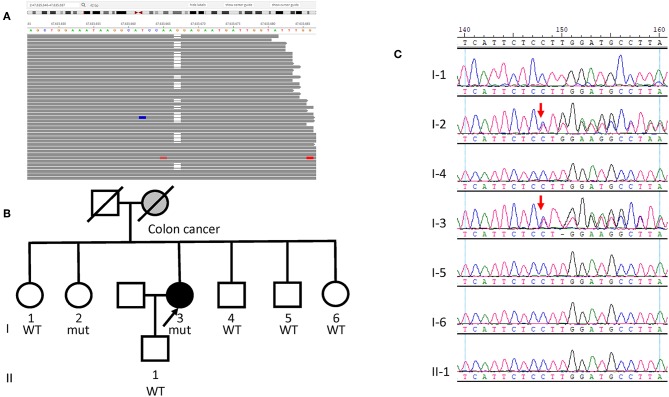
Germline *MSH2* mutation identified in Case 1. **(A)** Sequencing data visualized with Integrated Genome Viewer (Broad Institute) shows the *MSH2* variant (NM_000251.2, c.340delG, p.E114Rfs^*^60). Blank column denotes the deletion of a nucleotide G. **(B)** Pedigree of the family of Case 1. The proband is indicated with an arrow and gray denotes manifestation of colon cancer. **(C)** Sanger sequencing of the *MSH2* c.340delG mutation in the family shows the sister (I-2) also harbors the variant. Red arrows indicate the presence of mutation.

Case 1 is a 62-year-old female never-smoker with newly diagnosed lung adenocarcinoma. Her mother had colon cancer in her early 40s. Her father and five siblings had no history of cancer. The patient underwent a resection of a single polyp at the age of 60. Final pathology showed tubular adenoma. She also had a stable breast nodule at age 62, and no medical intervention was performed until her diagnosis of lung adenocarcinoma. At age 62, a CT scan showed a 24.2 × 22.5 mm nodule in the left upper lung during her regular physical examination, and later an MRI showed multiple lesions on the left occipitoparietal lobe and cerebellum. A tissue biopsy demonstrated lung adenocarcinoma that stained positive for TTF-1, and CK7, but was CK5-negative. As the tissue biopsy did not provide an adequate amount of DNA for NGS, peripheral blood samples were sent for NGS liquid biopsy. Somatic missense mutations in *MAP2K2* (NM_030662.3, c.1069C>T, p.R357W) and *GNAS* (NM_080425.2, c.1856G>A, p.C619Y) were detected in ctDNA with variant allele fractions (VAFs) of 0.005 and 0.004, respectively. A germline heterozygous loss-of function variant in *MSH2* (NM_000251.2, c.340delG, p.E114Rfs^*^60) was also detected (Figure 2A). This mutation is a frameshift mutation that is likely to cause partial or complete loss of the gene product. It has never been previously reported and was absent from controls in the NHLBI GO Exome Sequencing Project (https://evs.gs.washington.edu/EVS/), 1000 Genomes Project (11) and Exome Aggregation Consortium (http://exac.broadinstitute.org). Other pathogenic frameshift mutations 5' to this position were reported in cancer patients (12, 13). As a result, it was classified as “likely pathogenic” according to the American College of Medical Genetics and Genomics (ACMG) guideline. As stated above, IHC showed intact expression of MSH2, MSH6, MLH1, and PMS2 (Figure S1A). PCR-based MSI testing was not done due to insufficient tissue availability. As no actionable somatic mutations were identified and TMB was low, the patient underwent first line platinum based chemotherapy, and achieved the best response of stable disease. Due to the diagnosis of Lynch syndrome, all of the patient's siblings and her son were tested for the *MSH2* p.E114Rfs^*^60 mutation. The patient's sister also harbored the variant and was diagnosed with Lynch syndrome (Figures 2B,C).

**Figure 3:**
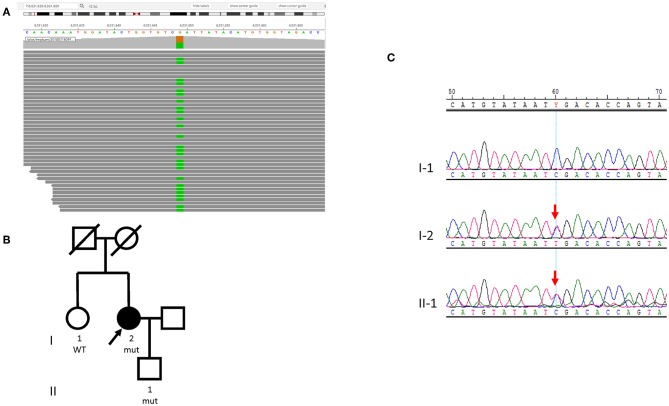
Germline *PMS2* mutation identified in Case 2. **(A)** Sequencing data visualized with Integrated Genome Viewer (Broad Institute) shows the *PMS2* variant (NM_000535.5, c.943C>T, p.R315^*^). The column with green color indicates the substitution of a nucleotide G with A (*PMS2* is encoded by the reverse strand). **(B)** Pedigree of the family. The proband is indicated with an arrow. **(C)** Sanger sequencing of the *PMS2* c.943C>T mutation in the family shows the son (II-1) also harbors the variant. Red arrows indicate the presence of mutation.

Case 2 is a 74-year-old female never-smoker with newly diagnosed lung adenocarcinoma. She had multiple solid nodules in both lungs that had been stable for 5 years until March 2017, when gradual elevation of carcinoembryonic antigen was noted during a regular physical examination. She had no family history of lung cancer. In March 2017, a tissue biopsy showed lung adenocarcinoma that was TTF-1+, CK7+, CK5-. Similar to Case 1, this patient's tissue biopsy was inadequate for NGS testing, and peripheral blood samples were sent for NGS liquid biopsy. A somatic *PTCH1* (NM_000264.3 c.2321G>T, p.G774V) mutation was detected in ctDNA with a VAF of 0.005. A germline heterozygous nonsense mutation was identified in *PMS2* (NM_000535.5, c.943C>T, p.R315^*^, Clinvar ID: 91382) (Figure 3A). This particular variant has been reported in individuals affected with Lynch syndrome and colon cancer (14–18), and we diagnosed the patient with Lynch syndrome. IHC was performed for MSH2, MSH6, MLH1, and PMS2, and all four proteins showed intact expression (Figure S1B). PCR-based MSI testing of the tissue revealed the tumor was MSS (Figure S2). As no actionable somatic mutations were identified and TMB was low, the patient initially refused chemotherapy, and gefitinib was tried as first line therapy for 2 months with the best response of stable disease. The patient then switched to platinum based chemotherapy. The patient was referred to genetic counseling, where her sister and son underwent germline genetic testing for the *PMS2* p.R315^*^ mutation. Her sister did not have the pathogenic mutation. However, this germline mutation was found in her son (Figures 3B,C).

## Discussion

To our knowledge, this represents the first large-scale retrospective study to date to thoroughly investigate the relationship among lung cancer, MMR deficiency, and Lynch syndrome. Our investigation of 1,179 lung cancer patients showed six cases with pathogenic or likely-pathogenic germline mutations in *PMS2, MSH2*, or *MSH6*, leading to the diagnosis of Lynch syndrome in these six individuals. Lynch syndrome-related cancers frequently arise through somatic “second hits” that inactivate MMR (19, 20). We therefore evaluated if the lung cancers in the six Lynch syndrome patients in our sample set were associated with the underlying germline MMR mutations, or instead arose sporadically. In our study, no somatic MMR mutations were identified in these patients (Table 1). None of them exhibited loss of MMR protein expression, or MSI, and none of them had elevated TMBs. Furthermore, the age of lung cancer onset in these six Lynch syndrome patients was older than typical hereditary cancers. Therefore, we speculate that, unlike typical Lynch syndrome-related cancers, the lung cancers in these patients were not related to the deleterious germline mutations in MMR genes. This notion is consistent with previous reports that the incidence of MSI is very low in lung cancer (21, 22). Our findings indicate that lung cancers can develop sporadically (not associated with Lynch syndrome) and be the first presenting cancer in Lynch syndrome patients. This is an important consideration for clinicians routinely performing NGS, as such investigations can result in independent Lynch syndrome diagnosis unrelated to the presenting malignancy, provided germline variants are properly examined.

Recent developments in cancer immunotherapy have resulted in an increased clinical importance of MMR deficiency/MSI in cancer patients. Specifically, the FDA recently approved pembrolizumab for treatment of patients with advanced pan-solid tumors with MSI or MMR deficiency (23, 24). None of Lynch syndrome patients in our study displayed MSI or MMR deficiency. This suggests that for lung cancer patients with Lynch syndrome, the utility of MSI, or MMR deficient guided immunotherapy is limited since these cases are likely sporadic. However, larger studies are needed to confirm this finding. These results must also be interpreted with caution, as our cohort consisted of mainly Chinese patients with non-small cell lung cancer.

While the detection of (rare) MMR gene mutations may be of importance for additional therapeutic options for some cancer patients, it can also be beneficial to the relatives of the tested patients. We were able to identify new Lynch syndrome cases in the first degree relatives of lung cancer patients with germline MMR gene mutations (the sister of Case 1 and the son of Case 2). This can result in appropriate cancer screen and surveillance for these individuals, and hopefully early detection of any malignancies that might develop. The relatives of these patients would likely not otherwise be screened based on the standard ACMG genetic screening algorithm. Our study suggests that practicing oncologists should be aware of the possibility of detecting clinically significant germline variants in cancer patients when using targeted paired tumor-normal NGS, even when the primary goal is to identify actionable somatic mutations. The additional germline variant information collected in these assays may facilitate screening and preventive measures for secondary cancers, and have implications for identifying cancer risks in relatives.

In summary, sporadic lung cancer may occur in Lynch syndrome patients. Paired tumor-normal targeted NGS in clinical practice can be used to detect undiscovered hereditary syndromes in that may or may not be related to the cancer patient's primary diagnosis. Together, comprehensive NGS provides crucial therapeutic guidance for cancer management, as well as hereditary syndrome screening helping to reduce risk for patients and their families. These measures should be integrated into the precision care of lung cancer patients.

## Methods

### Patients and Samples

This study retrospectively enrolled 1,179 patients with lung cancer who underwent genomic profiling with a hybridization capture-based NGS assay (Geneplus-Beijing, Beijing, China) from January 1, 2017, through May 31, 2018. Paired tumor or cell free DNA and peripheral blood samples were used to identify actionable mutations for targeted therapy for these patients with advanced lung cancer. All patients provided written informed consent to perform germline mutation analysis. This study was performed under a protocol approved by the Institutional Review Board of Geneplus-Beijing.

### DNA Extraction, Targeted Capture, and Next-Generation Sequencing

Genetic analysis was conducted as previously described (25, 26). Briefly, ctDNA was isolated from 4 to 5 mL of plasma using the QIAamp Circulating Nucleic Acid Kit (Qiagen, Valencia, CA). Serial sections from formalin-fixed paraffin-embedded tumor tissues were used for genomic tumor DNA extraction using the QIAamp DNA mini kit (Qiagen, Valencia, CA). DNA from leukocytes was extracted using the DNeasy Blood Kit (Qiagen, Valencia, CA). Sequencing libraries were prepared from ctDNA using KAPA DNA Library Preparation Kits (Kapa Biosystems, Wilmington, MA, USA), and genomic DNA sequencing libraries were prepared with Illumina TruSeq DNA Library Preparation Kits (Illumina, San Diego, CA). Libraries were hybridized to custom-designed biotinylated oligonucleotide probes (Roche NimbleGen, Madison, WI, USA) targeting 1,021 genes (Supplementary Table S1), including *MLH1, MSH2, MSH6*, and *PMS2*. Prepared libraries were sequenced on a NextSeq CN 500 (Illumina, San Diego, CA).

### Sequencing Data Analysis and Variant Interpretation

Sequencing data was analyzed using default parameters. Adaptor sequences and low-quality reads were removed. The clean reads were aligned to the reference human genome (hg19) with Burrows-Wheeler Aligner (BWA; version 0.7.12-r1039). Variants were called with GATK (version 3.4-46-gbc02625) and MuTect (version 1.1.4). Contra (v2.0.8) was used to detect copy number variants, and BreakDancer (v1.4.5) was used to detect structural variants. The final candidate variants were all manually verified using Integrative Genomics Viewer (25, 26). Targeted capture sequencing required a minimal mean effective depth of coverage of 300 × in tissues and 1,000 × in plasma samples, and for the 6 patients with MMR germline mutations, the mean effective depth of coverage is 1,336 × in tissues and 1,102 × in plasma samples and 304 × in germline DNA samples (Supplementary Table S4). Variants were filtered to exclude synonymous variants, known germline variants in dbSNP, and variants that occur at a population frequency of >1% in the Exome Sequencing Project. Germline variants were interpreted following ACMG guideline, and the variants were classified into pathogenic, likely pathogenic, unknown significance, likely benign, and benign.

### TMB Evaluation

TMB was calculated as the number of somatic non-synonymous single-nucleotide variants and small insertions/deletions per Mb in the coding region (with variant allele fraction ≥0.03 for tissue, and ≥0.005 for peripheral blood, respectively). TMB-high patients were identified with ≥9 muts/MB using the top quartile threshold of 2,000 samples from the Geneplus database (9, 10).

### MMR Protein Immunohistochemistry

Biopsy tissues were formalin-fixed and stained with Hematoxylin & Eosin and the following antibodies: MHL1 (MAB-0642, Fuzhou Maxin, China), MSH2 (MAB-0291, Fuzhou Maxin, China), MSH6 (MAB-0643, Fuzhou Maxin, China), and PMS2 (MAB-0656, Fuzhou Maxin, China), as previously described (27).

### MSI Analysis

The MSI status of tumor tissue samples using NGS was inferred using MSIsensor (v0.5), which reported the percentage of unstable somatic microsatellites through Chi-square test on predefined microsatellite regions covered by our panel. Default parameters were used (4), and MSI-high was defined on an empirically defined cutoff of MSI score>8% (8). PCR based MSI detection was performed as described previously using a multiplex PCR system and capillary electrophoresis (28).

### Statistical Analysis

This is a retrospective study with case presentation. Patient demographics were summarized by age, gender, pathology subtype, clinical stage, and smoking history. Age was described by median with range, and the rest were described by number of cases and percentage. The age and TMB comparison was done by two-tailed Mann Whitney test with the differences considered significant when *P* < 0.05 (Graphpad Prism 6).

## Data Availability

The raw data supporting the conclusions of this manuscript will be made available by the authors, without undue reservation, to any qualified researcher.

## Ethics Statement

This study was performed under a protocol approved by the Institutional Review Board of Geneplus-Beijing. Two patients presented as cases in this study agreed and provided written informed consent. Written informed consent was obtained from the patients for publication of their clinical data and any accompanying images.

## Author Contributions

RC, KH, and RG: conceptualization. SS, YL, and AE: methodology and validation. SS, YL, AE, FZ, SJ, WG, TY, LC, WW, WC, and MY: formal analysis. KH and RG: investigation. KH and RG: resources. SS, YL, and AE: data curation. SS and YL: writing—original draft preparation. AE, WC, MY, RC, and KH: writing—review and editing. All the authors have approved the final manuscript.

### Conflict of Interest Statement

MY and RC are consultants for Geneplus-Beijing Institute. The remaining authors declare that the research was conducted in the absence of any commercial or financial relationships that could be construed as a potential conflict of interest.
