# Supplementary material for: Identification of Germline Mismatch Repair Gene Mutations in Lung Cancer Patients With Paired Tumor-Normal Next Generation Sequencing: A Retrospective Study
Source: Front Oncol. 2019 Jun 26;9:550. doi: 10.3389/fonc.2019.00550 (PMC6607931; doi:10.3389/fonc.2019.00550)
Supplement: Supplementary file 2 [file Data_Sheet_1.docx]

Supplementary Material

# Supplementary Figures


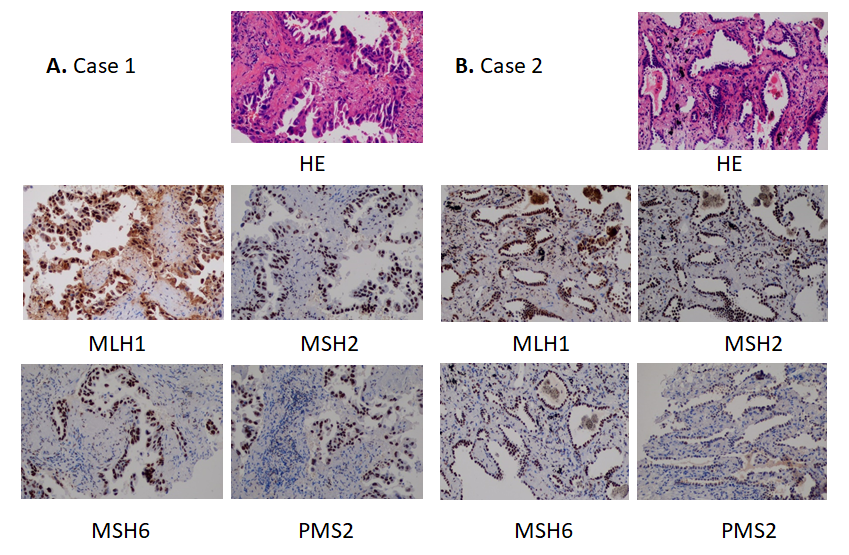


**Figure S1.** HE and IHC staining of MLH1, MSH2, MSH6 and PMS2 of lung cancer tissues from case 1 (A), and case 2 (B) (200x magnification).

**
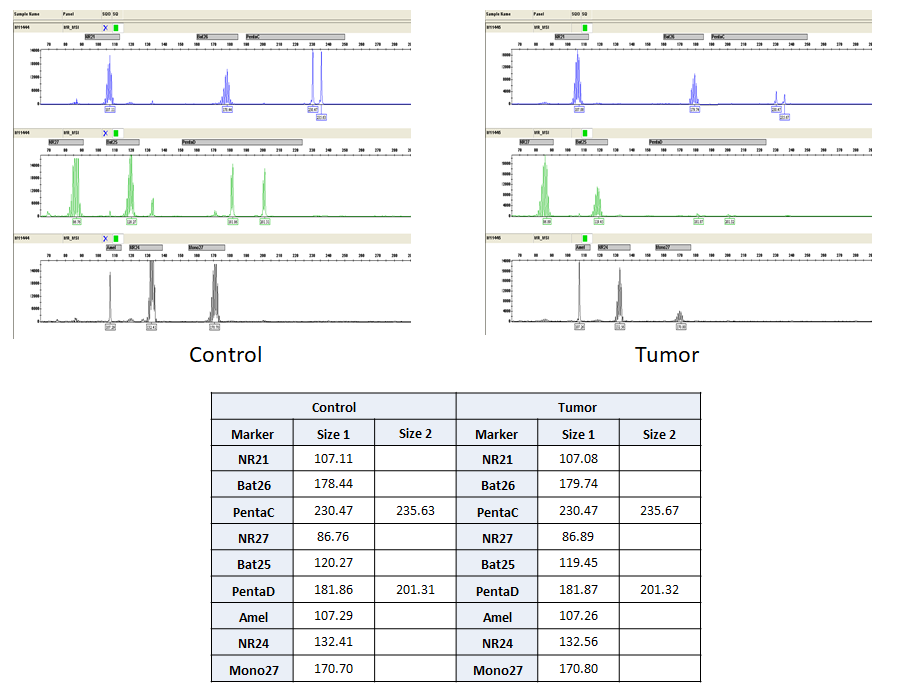
**

**Figure S2.** Microsatellite instability (MSI) profiles of control and tumor samples (Upper panel). No shifts of microsatellite repeat lengths were noticed indicating the MSS statue. Microsatellite repeat lengths are listed in the table (Lower panel).
